# Development of Alginate Hydrogels Incorporating Essential Oils Loaded in Chitosan Nanoparticles for Biomedical Applications

**DOI:** 10.3390/molecules29225318

**Published:** 2024-11-12

**Authors:** Ioanna Pitterou, Flora Kalogeropoulou, Andromachi Tzani, Konstantinos Tsiantas, Maria Anna Gatou, Evangelia Pavlatou, Anthimia Batrinou, Christina Fountzoula, Anastasios Kriebardis, Panagiotis Zoumpoulakis, Anastasia Detsi

**Affiliations:** 1 Laboratory of Organic Chemistry, Department of Chemical Sciences, School of Chemical Engineering, National Technical University of Athens, 15772 Athens, Greece; ipitterou@mail.ntua.gr (I.P.); florakalo@gmail.com (F.K.); atzani@mail.ntua.gr (A.T.); 2 Laboratory of Chemistry, Analysis and Design of Food Processes, Department of Food Science and Technology, University of West Attica, 12243 Athens, Greece; ktsiantas@uniwa.gr (K.T.); batrinou@uniwa.gr (A.B.); pzoump@uniwa.gr (P.Z.); 3 Laboratory of General Chemistry, School of Chemical Engineering, National Technical University of Athens, 15772 Athens, Greece; mgatou2@mail.ntua.gr (M.A.G.); pavlatou@chemeng.ntua.gr (E.P.); 4 Laboratory of Chemistry, Biochemistry and Cosmetic Science, Department of Biomedical Sciences, School of Health and Caring Sciences, University of West Attica, 12243 Athens, Greece; chfountz@uniwa.gr; 5 Laboratory of Reliability and Quality Control in Laboratory Hematology, Department of Biomedical Sciences, School of Health and Caring Sciences, University of West Attica, 12243 Athens, Greece; akrieb@uniwa.gr

**Keywords:** chitosan nanoparticles, alginate hydrogels, essential oils, antimicrobial activity, NADES, green nanotechnology

## Abstract

A hybrid alginate hydrogel–chitosan nanoparticle system suitable for biomedical applications was prepared. Chitosan (CS) was used as a matrix for the encapsulation of lavender (*Lavandula angustifolia*) essential oil (LEO) and Mentha (*Mentha arvensis*) essential oil (MEO). An aqueous solution of an acidic Natural Deep Eutectic Solvent (NADES), namely choline chloride/ascorbic acid in a 2:1 molar ratio, was used to achieve the acidic environment for the dissolution of chitosan and also played the role of the ionic gelator for the preparation of the chitosan nanoparticles (CS-NPs). The hydrodynamic diameter of the CS-MEO NPs was 130.7 nm, and the size of the CS-LEO NPs was 143.4 nm (as determined using Nanoparticle Tracking Analysis). The CS-NPs were incorporated into alginate hydrogels crosslinked with CaCl_2_. The hydrogels showed significant water retention capacity (>80%) even after the swollen sample was kept in the aqueous HCl solution (pH 1.2) for 4 h, indicating a good stability of the network. The hydrogels were tested (a) for their ability to absorb dietary lipids and (b) for their antimicrobial activity against Gram-positive and Gram-negative foodborne pathogens. The antimicrobial activity of the hybrid hydrogels was comparable to that of the widely used food preservative sodium benzoate 5% *w*/*v*.

## 1. Introduction

Biocompatible nanosystems and materials are currently gaining ground as potential tools for biomedical applications. Enhancement of the green character of nanosystems is a very important issue that can be addressed by taking several actions. For example, the use of biodegradable and non-toxic natural carbohydrates, such as chitosan, alginate, cellulose, etc., as matrices for the encapsulation of bioactive compounds and/or natural products, along with the fact that the biopolymers are obtained from renewable sources or from biomass valorization, confers a greener character to the nanosystems, thus making a step towards more sustainable and safer materials. In addition, the use of toxic solvents and additives during the preparation of the nanosystems can be confined, and greener alternatives should be considered.

Hydrogels are 3D hydrophilic polymeric networks that are capable of swelling in water. In the last few years, there has been increasing interest in these materials thanks to their solid and liquid-like properties, high biocompatibility, easy preparation, and versatile applications [1]. Nanoparticles incorporated in hydrogels have recently emerged as a hybrid nanosystem, which combines the advantages of both materials in one formulation. This combination can help manage several disadvantages of the nanoparticles (such as toxicity and uncontrolled release of the encapsulated compounds), can modify the mechanical and swelling properties of the hydrogels, and provides more options for administration [2,3,4,5,6,7].

Essential oils (EOs), the multi-component mixtures obtained from aromatic plants comprising mainly of low molecular weight metabolites such as terpenoids, phenolic compounds, and aliphatic components, possess a diverse array of biological activities, including antibacterial, antioxidant, antifungal, and antiviral properties [8]. EOs are widely used in food preparations, mainly as natural preservatives and antimicrobials. Recent research results indicate that EOs can also be effective in body weight control and can play a role in the prevention of disorders related to metabolic syndrome [9,10].

In the frame of our ongoing studies on the development of nanosystems and hydrogels of enhanced bioactivity and biocompatibility, we proceeded to prepare an alginate hydrogel in which chitosan nanoparticles encapsulating lavender essential oil (*Lavandula angustifolia*) (LEO) and Mentha essential oil (*Mentha arvensis*) (MEO) were incorporated. The final hydrogels were tested (a) for their ability to absorb dietary lipids and (b) for their antimicrobial activity.

Alginate was selected as it is one of the most widely and successfully used natural polysaccharides for the preparation of nanosystems and hydrogels with food and biomedical applications. Alginate is an anionic, hydrophilic biopolymer with proven antioxidant, anti-inflammatory, and antimicrobial activity. In addition, this biopolymer is an efficient blood glucose modulator and is used as a dietary supplement for weight management [11,12].

Chitosan was chosen as the encapsulation matrix as it is a natural polysaccharide that is non-toxic, biodegradable, and biocompatible with the human digestive system. The cationic nature of chitosan leads, under acidic conditions, to the development of various forms, such as nano/microparticles, emulsions, fibers, hydrogels, films, and membranes. Chitosan is commonly used as a food supplement and is frequently employed to regulate weight by preventing the absorption of dietary fats [13,14].

Chitosan is not soluble in water, and it can only be dissolved in aqueous acidic solutions, usually using acetic acid or other organic or inorganic acids. In our previous work, we have proven the ability of aqueous solutions of the acidic Natural Deep Eutectic Solvents (NADES) choline chloride/lactic acid (molar ratio ChCl: LA 1:1.5) and betaine/lactic acid (molar ratio bet: LA 1:2) to efficiently dissolve chitosan. Applying the casting method, chitosan films were prepared from these solutions without the need to add plasticizers [15]. Based on these encouraging results, we decided to investigate the ability of a NADES comprised of choline chloride/ascorbic acid in a 2:1 molar ratio (ChCl/AA) to (a) dissolve chitosan and (b) act as an ionic gelator in order to avoid the use of sodium tripolyphosphate (TPP) in the process of the formation of nanoparticles to encapsulate the chosen essential oils. Taking a step forward, we proceeded to incorporate the nanoparticle dispersion in alginate hydrogels in order to prepare a hybrid nanosystem with potential biomedical applications.

## 2. Results

The present study focused on the potential of the ChCl/AA NADES to effectively act as dissolution and crosslinking agent for the formation of LEO or MEO-loaded CS-NPs as well as the incorporation of the CS NPs in alginate hydrogels to produce new materials with (a) the ability to absorb dietary lipids and (b) antimicrobial activity.

In order to prepare the chitosan nanoparticles, the polymer has to be dissolved in an aqueous acidic solution (acetic acid, hydrochloric acid, formic acid, and lactic acid are the most commonly used acids for this purpose) so that the amino groups of chitosan are protonated, and the polymer becomes a positively charged polyelectrolyte. In our recent study on the preparation of chitosan films using NADES, we have shown that chitosan of low molecular weight was easily dissolved in aqueous solutions of acidic NADESs 1% *w*/*v* [16]. The NADES choline chloride/D,L-lactic acid 1:1.5 (molar ratio) and betaine/D,L-lactic acid 1:2 (molar ratio) were successfully used for this purpose.

The present research was dedicated to the encapsulation of LEO and MEO in CS-NPs. Therefore, we decided to explore the ability of NADES to act (a) as the dissolution agent for chitosan and (b) as the ionic gelator for nanoparticle formation in order to replace TPP. To achieve these goals, the components of the NADES were selected based on their safety regarding pharmaceutical and food applications as well as on the basis that, when dissolved in water, the NADES results in an acidic solution, which is an indispensable requirement for chitosan dissolution. The selected NADES is composed of choline chloride [16] and ascorbic acid (Vitamin C), both compatible with food applications while possessing also antimicrobial activity. The idea is to form added-value CS-NPs, as the presence of the NADES components in the nanoparticles, apart from chitosan dissolution and gel formation, may contribute to the desired bioactivity.

### 2.1. Characterization of NADES

#### NMR Analysis

**Figure 1:**
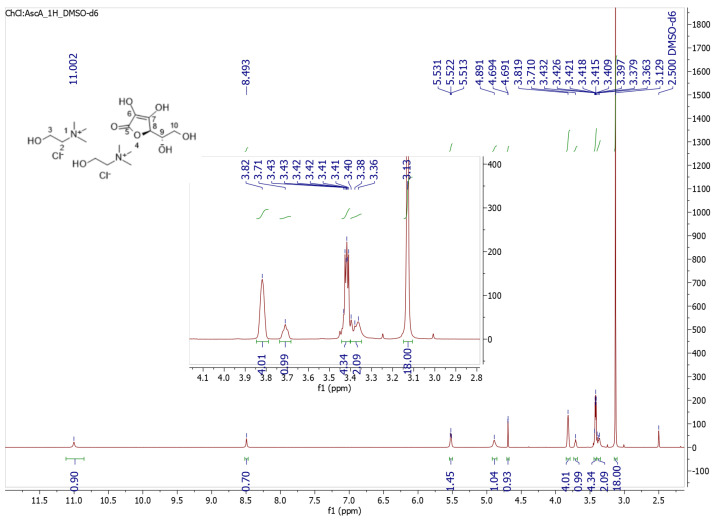
^1^H NMR (DMSO-d_6_) spectrum of choline chloride–ascorbic acid (2:1) NADES.

The ^1^H NMR analysis on the synthesized NADES derived from choline chloride and ascorbic acid in a molar ratio of 2:1 was performed, and the spectrum is presented in Figure 1. The chemical shifts of the peaks related to the protons of ChCl appear at 3.82, 3.42, 3.13, and 5.55 ppm. More specifically, the broad peak at 3.82 ppm can be attributed to the two equivalent protons of the methylene at position 2 of the two molecules of ChCl, while the multiplet at 3.43–3.41 ppm is attributed to the methylene at position 3 of the ChCl molecules. At 3.13 ppm, there appears a single peak that corresponds to the protons of the three methyl groups of ChCl. Finally, the peak at 5.52 ppm corresponds to the protons of the hydroxyl group of ChCl. Regarding the chemical shifts of the protons of ascorbic acid, the protons of the hydroxyl groups of the heterocyclic ring appear as more deshielded at lower magnetic fields. Specifically, the single peaks at 11.00 ppm and 8.49 ppm can be attributed to the hydroxyl protons of positions 6 and 7, whereas the broad peak at 4.89 ppm corresponds to the two protons of the hydroxyl groups of the aliphatic chain of ascorbic acid. The H-8 of ascorbic acid gives a characteristic singlet at 4.69 ppm, while at 3.71 ppm appears a broad signal that is owed to H-9. Finally, the two protons of the methylene group of position 10 give a broad signal at 3.40–3.36 ppm.

The ^1^H NMR spectrum of choline chloride/ascorbic acid 2:1 NADES verifies the purity of the solvent as it lacks any signals that could be attributed to impurities or by-products formed during the synthesis process. The 2:1 molar ratio of choline chloride and ascorbic acid is also corroborated by the integration of the signals that correspond to each component. For example, the signal of the methylene protons of choline chloride at position 4 (Figure 1) appears at 3.82 ppm and integrates for 4H (two methylene groups, one from each choline chloride molecule), and the signal of the proton at position 8 of the ascorbic acid ring appears at 4.69 ppm and integrates for ^1^H.

### 2.2. Chemical Composition of Essential Oils

**Figure 2:**
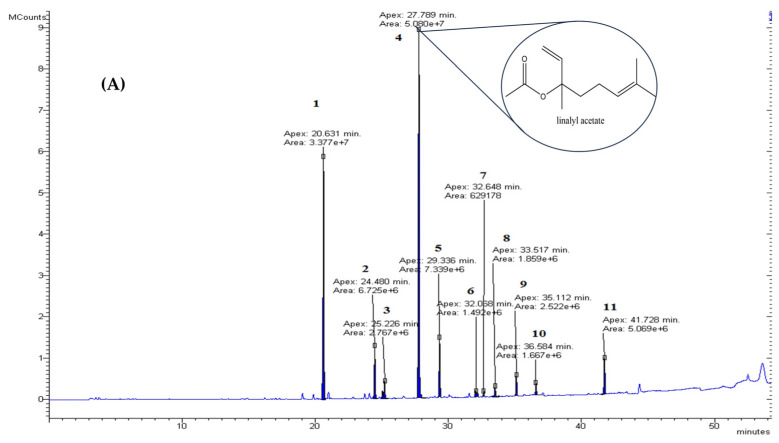

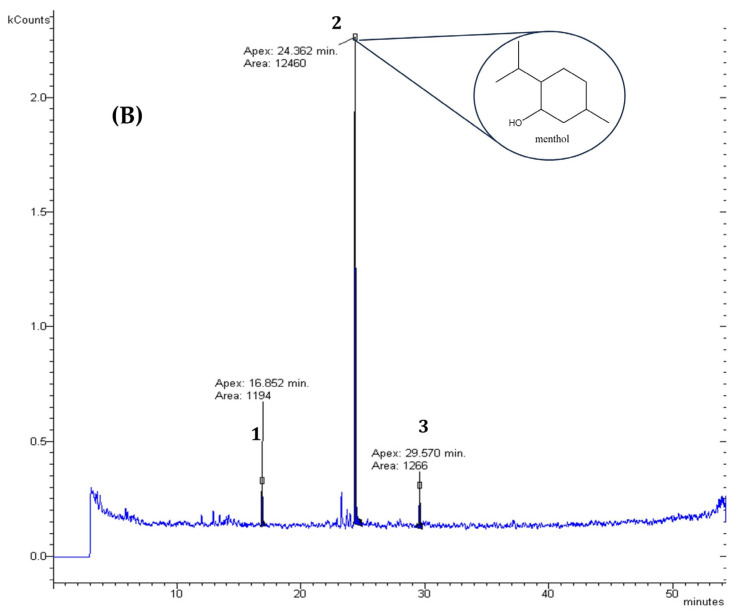
GC–MS chromatograms of the essential oils: (**A**): lavender (*Lavandula angustifolia*) essential oil; (**B**): Mentha (*Mentha arvensis*) essential oil.

The chemical composition of lavender (*Lavandula angustifolia*) and Mentha (*Mentha arvensis*) essential oils was determined by GC–MS. The components of the essential oils were identified by comparing the retention times, as well as the resulting mass spectra, with data from the electronic libraries available in the NIST MS Search software version 2.0. The GC–MS chromatograms of the essential oils are presented in Figure 2, while the analysis of their chemical composition is presented in Table 1. The main components identified in LEO were linalyl acetate, linalool, and terpinenol-4. In the MEO, the main component was menthol.

### 2.3. Characterization of CS NPs

#### 2.3.1. Dynamic Light Scattering (DLS)

The size, polydispersity index (PdI), and *ζ*-potential values for the formed CS-NPs are presented in Table 2.

The mean hydrodynamic diameter of the CS-MEO-NPs in aqueous dispersion is 303.7 nm, whereas for the CS-LEO-NPs, the value is 313 nm. The PdI values of both dispersions (0.381 and 0.410, respectively) indicate moderately uniform size dispersion. The obtained positive *ζ*-potential values indicate the formation of nanosystems adequately stable in aqueous dispersions. The positive charge is expected and owed to the protonated amino groups of chitosan.

In this work, the CS-NPs encapsulating MEO or LEO are formed using NADES ChCl/AA as the medium for the dissolution of CS as well as the crosslinking agent without the use of sodium tripolyphosphate (TPP) or any other gelator. It is interesting to note that the properties of the obtained essential oil-loaded CS-NPs are comparable with those reported in the literature for CS-NPs encapsulating essential oils prepared via ionic gelation using sodium tripolyphosphate (TPP) as the gelator. For example, Hadidi et al. report a mean hydrodynamic diameter of 444.5 nm, PdI 0.117, and ζ-potential +10.14 mV for CS-NPs encapsulating clove essential oil when the ratio of CS/essential oil was 1:1 (as in our study) [16]. In the work of Adel et al., the CS-NPs encapsulating *Mentha piperita* essential oil had a size of 178 nm and the ζ-potential was +32.2 mV [17], whereas Mondejar-Lopez et al. prepared CS-NPs encapsulating garlic essential oil, which had a mean hydrodynamic diameter of 253 nm, PdI > 0.7, and ζ-potential +19.8 mV [18]. Moreover, in the study of Granata et al., chitosan nanoparticles encapsulating oregano and thyme essential oils were prepared using the ionotropic gelation method. The resulting nanoparticle sizes are ~407 nm (oregano EO) and 449 nm (thyme EO), and PdI and ζ-potential values indicate good stability and uniformity of the nanoparticles [19].

Based on the above analysis, it can be concluded that the CS-NPs encapsulating MEO and LEO prepared via our proposed methodology, which uses a NADES as the gelator and solubilizing agent for chitosan, have characteristics that are comparable to the CS-NPs prepared using TPP as the gelator and aqueous CH_3_COOH to dissolve chitosan.

#### 2.3.2. Nanoparticle Tracking Analysis (NTA)

**Figure 3:**
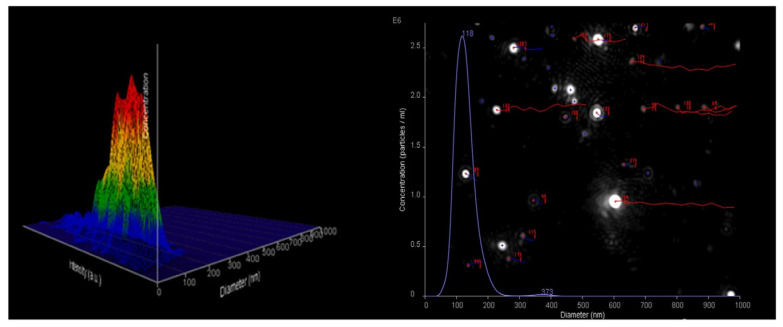
An indicative example of the size distribution profile generated by NTA.

The NTA technique demonstrates high specificity, linearity, accuracy, and precision, making it a reliable method for quantifying particle concentration and determining the hydrodynamic diameter. The results are shown in Table 3 and Figure 3.

According to the DLS analysis, the nanoparticles exhibit a size range of 303 to 313 nm. However, when using NTA, a smaller size range of 130 to 158 nm was measured. This difference can be attributed to the distinct principles and measurement methodologies employed by the two techniques. DLS calculates an average size measurement based on the intensity of scattered light, which can be influenced by larger particles present in the sample, resulting in a wider size distribution. Conversely, NTA tracks and measures individual nanoparticles, capturing their hydrodynamic diameter and providing a more precise representation of the size distribution [20,21].

#### 2.3.3. TEM Analysis

**Figure 4:**
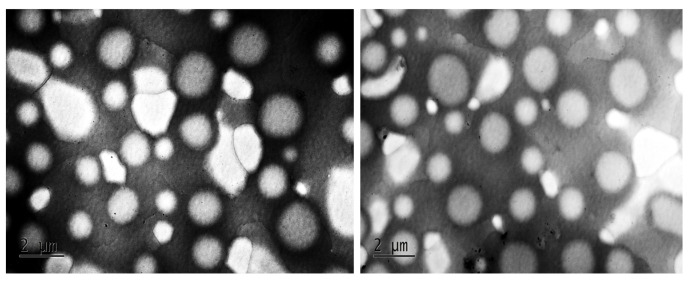
TEM image of synthesized CS NPs.

The TEM analysis of the synthesized CS-NPs provides accurate information about the shape, morphology, and size of the NPs. The TEM micrographs reveal that the NPs have a spherical shape, exhibiting smooth morphological characteristics. The sizes of the NPs range from less than 500 nm to 1 µm, as demonstrated in Figure 4.

#### 2.3.4. In Vitro Essential Oil Release Study

**Figure 5:**
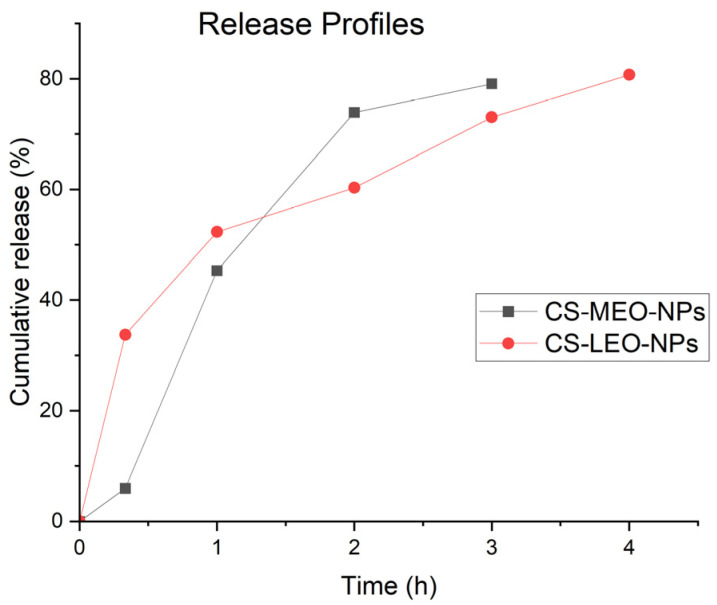
Release profile of MEO and LEO from the corresponding CS-NPs studied at 37 °C, pH 7.4.

The release profiles of the EOs from the CS-NPs were determined using GC–MS. The release profile of CS-MEO NPs exhibits two phases. Phase I occurred within the first 1 h and was characterized by a burst release effect during which 45% of menthol, the main component of MEO, was rapidly released. This effect was attributed to the proximity of the essential oil to the surface of the NPs. Phase II was characterized by a slower and sustained release due to the slow diffusion of the encapsulated essential oil from the CS-NPs in the aqueous medium. After 3 h, a cumulative release of 80% of menthol was observed (Figure 5). The release kinetics followed the Higuchi model, which was supported by a high correlation coefficient (R^2^). The values of the transport exponent (*n*) of the Korsmeyer–Peppas model suggested that the release mechanism was anomalous transport (Table 4).

An analogous release behavior was observed for the release of the components of LEO from the CS-LEO-NPs. Phase I was observed after 1 h, when approximately 50% of linalyl acetate was released. Phase II was characterized by a slower release, and after 4 h, a cumulative release of 80% of linalyl acetate was observed (Figure 5). The release kinetics followed the Higuchi model, whereas the values of the transport exponent (*n*) of the Korsmeyer–Peppas model suggested that the release mechanism was anomalous transport (Table 4).

### 2.4. Preparation of Alginate Hydrogels Incorporating the CS-MEO-NPs and CS-LEO-NPs and Study of Their Most Important Properties

The next step was the development of alginate hydrogels loaded with the prepared chitosan nanoparticles, using CaCl_2_ as the crosslinker. This was succeeded by adding the CS NPs dispersion in an aqueous solution of sodium alginate, followed by the dropwise addition of CaCl_2_ solution until a homogeneous hydrogel was formed. The hydrogels were then freeze-dried and stored in a desiccator to be studied further. Three hydrogels were prepared, one incorporating blank CS-NPs (without essential oil), one loaded with CS-MEO-NPs, and one loaded with CS-LEO-NPs.

One of the most important properties of hydrogels is their swelling behavior, which is related to the interaction between the polymer chains and water molecules, and it can be regarded as the first indication of the stability of the network. The swelling capacity and water retention of the prepared hydrogels were evaluated as described in the Experimental Section by immersing the hydrogels in a 0.1 M hydrochloric acid (HCl) solution (pH 1.2) at 37 °C. The results are presented in Table 5.

The swelling capacity of the alginate hydrogel incorporating CS-LEO-NPs is 407% in the first 10 min (Table 5). This hydrogel maintained a high water retention (approximately 100%) after 2 h (Table 5). Regarding the CS-MEO-NPs hydrogel (Table 5), it is observed that the swelling of the hydrogel reaches 496% in the first 10 min. The swelling continues to increase, and after 1 h, it reaches the highest value. The water retention is maintained at high levels (approximately 100%), as well. At 2 h, it is observed that the water retention reaches 99% of its initial value. These properties render the hydrogel suitable for potential applications in biological tissue replacement [22]. Finally, regarding the hydrogel with CS Blank-NPs, it is observed that at 10 min, the swelling capacity (Table 5) reaches 213% before reaching the equilibrium point, while the water retention (Table 5) is 100% and drops to 99% after 2 h.

The capacity of hydrogels to absorb dietary lipids holds significant importance for their application in food supplements [23]. Hydrogels with high lipid absorption capability offer a promising solution for controlling fat intake and enhancing the nutritional profile of food products. By efficiently sequestering lipids, these hydrogels can aid in reducing the caloric content of food supplements, making them more suitable for individuals seeking to manage their weight or adhere to specific dietary requirements. Therefore, understanding and optimizing the capacity of the prepared hydrogels to absorb dietary lipids is crucial for developing functional food supplements with enhanced nutritional benefits [24,25].

The capacity of the prepared hydrogels to interact with three different types of dietary lipids from vegetable oils, namely sunflower oil, corn oil, and olive oil, was determined using an experimental model simulating the pH of the stomach and duodenum tract as described by Rodiguez et al. [25]. The vegetable oils tested were selected because they are the ones mostly used in Mediterranean and European diets, and they have differences in their content of fatty acids, triacylglycerides, phytosterols, and polyphenols [26].

The results of this study (Table 5) show that the alginate hydrogel incorporating the blank CS-NPs showed higher absorption capacity for the dietary lipids from sunflower and corn oil (1.5%) and lower for olive oil (0.95%). The incorporation of CS-NPs encapsulating MEO and LEO in the alginate hydrogels resulted in a significant increase in their ability to absorb dietary lipids from all the vegetable oils tested.

The alginate hydrogel loaded with CS-LEO-NPs showed the highest absorption capacity for sunflower oil lipids (5.6%) among all the hydrogels tested. The ability of the hydrogels loaded with CS-LEO-NPs and CS-MEO-NPs to absorb corn oil lipids was comparable (7.0% and 7.1%, respectively), as was the case for olive oil lipids (3.7% and 4.8%, respectively). Overall, it seems that the presence of chitosan nanoparticles loaded with the essential oils leads to an enhancement of the ability of the prepared hydrogels to absorb dietary lipids by three or four times in all cases, as compared to the hydrogel containing the blank chitosan nanoparticles. This observation leads to the hypothesis that there is a kind of synergistic effect that comes into play when the LEO and MEO are present in the formulation. The exact way that this phenomenon is expressed is worthwhile for further studies.

### 2.5. Optimization of the Preparation Process of the Alginate Hydrogel Incorporating CS-LEO-NPs

The alginate hydrogel incorporating CS-LEO-NPs showed the best-combined ability to absorb dietary lipids from the three vegetable oils tested. Thus, an optimization of the preparation process for this hydrogel was performed by implementing a Box–Behnken experimental design.

Key parameters for the preparation of the hybrid alginate hydrogel–chitosan nanoparticle system were selected to be studied as independent values for the experimental design. Specifically:

Factor A: The concentration of NADES (% *v*/*v*) used to prepare the nanoparticles. Factor A is an important parameter for the chitosan nanoparticle preparation, possessing a dual role in the process. NADES is used to dissolve chitosan and, at the same time, acts as an ionic gelator for the formation of the chitosan nanoparticles. The boundaries of the system for factor B were set with a minimum concentration of 2% *v*/*v* and a maximum of 5% *v*/*v*.

Factor B: The concentration of chitosan (% *w*/*v*) used to prepare the chitosan nanoparticles. This factor is important to be studied since the chitosan NPs in the hybrid hydrogel system can affect the hydrogel’s mechanical properties and swelling behavior.

Moreover, both factors, A and B, are crucial factors to be studied since both can significantly affect the size of NPs, which is a key parameter since the nanoparticle sizes [27] can influence the NP’s dispersion within the hydrogel and their interaction with biological systems. According to the literature, lower concentrations of chitosan lead to smaller particle sizes [28], which is desirable for most biomedical applications. Moreover, smaller particle size is desirable since, according to Agustiani et al., it can provide sustained delivery [29]. For these reasons, concentrations lower than 1% *w*/*v* were selected to be studied.

Factor C: The concentration of sodium alginate (% *w*/*v*) used to prepare the hydrogel. This factor may affect the network’s structure and, consequently, the hydrogel’s ability to absorb water and swell. The boundaries of the system for this factor were set with a minimum concentration of 1% *w*/*v* and a maximum of 5% *w*/*v*. These concentrations were selected since they are within the range that is usually applied for making hydrogels suitable for various applications [30,31,32].

As responses for the experimental design, the swelling ratio % (R1), the water retention ratio % (R2), and the oil absorption capacity % (R3) were selected.

A total of 15 experiments were conducted using the Box–Behnken experimental design, as shown in Table 6. To estimate the actual error, three center-point replications were chosen. Following the collection of response data, a statistical analysis was performed on the values and a mathematical model was extracted to accurately describe each response.

#### 2.5.1. Swelling Ratio

To study this response (R1), the Reduced Cubic Model was chosen. Upon developing a mathematical model that incorporated all parameters, statistically significant outcomes were observed, with a model F-value of 9.72 and *p*-value of 0.0110 (<0.005), indicating a 2.6% chance that the variance between the studied variables could be attributed to experimental errors or noise. To enhance the model’s performance, certain variables with high *p*-values were eliminated (AC, C^2^, ABC, A^2^C, AC^2^, B^2^C, BC^2^, A^3^, B^3^, C^3^), and their impact on the model was not evaluated.

The regression model that establishes a relationship between the process design parameters A (NADES % *v*/*v*), B (Chitosan % *w*/*v*), and C (Alginate % *w*/*v*), and the response variable of % swelling ratio (R1) is presented at the following Equation (1):

(1) $$\begin{array}{l} R1=415.56-370.83A-217.37B+62.52C+1427.04AB-86.95BC+82.56A^{2}-2737.59B^{2}-\\ 314.14A^{2}B+656.57{\mathrm{AB}}^{2} \end{array}$$

The values R^2^ and adjusted R^2^ are 0.95 and 0.85, respectively, which means that they are in reasonable agreement, indicating a good fit between the experimental and the predicted values. In addition, the statistical analysis shows a signal-to-noise ratio of 9.40, which indicates sufficient signal, and thus, this model can be used to navigate the design space. From the results above, it is clear that among the three parameters, chitosan concentration (% *w*/*v*) is the factor that contributes the most to the swelling ratio. The statistical significance of each variable is presented in Table 7.

According to the equation above, the amount of chitosan in combination with the amount of NADES (factor AB^2^) shows a statistically significant (*p* < 0.005) effect on the swelling capacity (SR) of the hydrogels.

**Figure 6:**
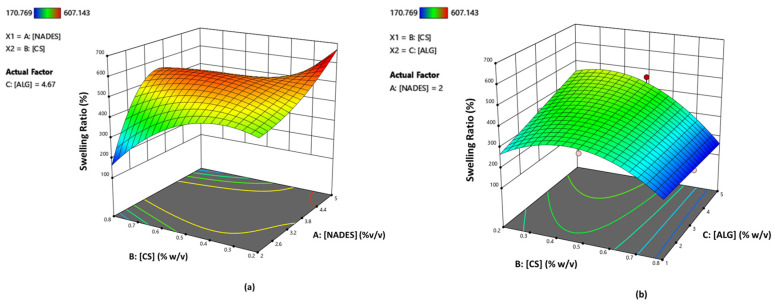
RSM plots for swelling ratio (%) response (R1) showing the correlation between the swelling ratio and the factors: (**a**) CS (% *w*/*v*) and NADES (% *v*/*v*) (alginate = 4.67% *w*/*v*) (**b**) alginate (% *w*/*v*) and CS (% *w*/*v*) (NADES = 2% *v*/*v*).

The 3D surface response plots illustrate the correlation between the analyzed factors and the chosen response variable (Figure 6a).

According to the RSM plots (Figure 6a), when keeping constant the parameter C (alginate % *w*/*v*) at 4.67% *w*/*v*, the maximum swelling capacity is facilitated by the use of higher amounts of NADES (higher than 4.4% *w*/*v*) in combination with lower percentages of CS (lower than 0.3% *w*/*v*). Additionally, regarding the correlation between parameters B and C, in order for higher swelling ratios to be achieved, higher percentages of alginate (>4% *w*/*v*) while lower amounts of CS (<0.3% *w*/*v*) are required (Figure 6b).

#### 2.5.2. Water Retention Ratio

To study the water retention ratio response, the Reduced Cubic Model was also chosen as the most appropriate. The F-value of the model is 10.25, which indicates that it is significant. To improve the model’s efficacy, certain variables with high *p*-values were excluded (BC, B^2^, C^2^, ABC, AB^2^, AC^2^, B^2^C, BC^2^, A^3^, B^3^, C^3^), and their influence on the model had not been assessed. Consequently, several interactions were eliminated, resulting in a statistically significant equation (model *p*-value 0.0054). The model exhibits an R^2^ of 0.93. The predicted R^2^ of 0.88 is in reasonable agreement with the adjusted R^2^ of 0.84, indicating a strong fit of the experimental values to the predicted values. The statistical analysis reveals a signal-to-noise ratio of 10.72, indicating sufficient signal and that this model can be used to navigate the design space.

The statistical significance of each variable is presented in Table 8.

The following Equation (2) shows the regression model that establishes a correlation between the process design parameters A, B, and C and the response variable of % water retention ratio (R2):

R2 = 183.84 − 80.60A − 105.43B + 10.95C + 93.75AB − 5.29AC + 10.90A^2^ − 15.20A^2^B + 1.01A^2^C (2)

Alginate, chitosan, and its combination with NADES show a statistically significant (*p* < 0.005) effect on the water retention capacity (WRR) of the hydrogels.

**Figure 7:**
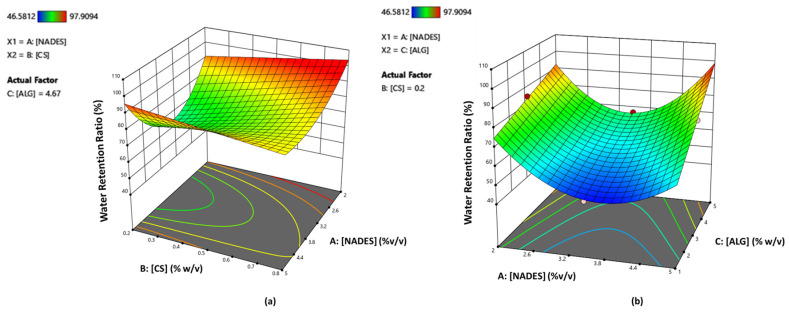
RSM plots for water retention ratio (%) response showing the correlation between water retention ratio and the factors: (**a**) CS (% *w*/*v*) and NADES (% *v*/*v*) (alginate = 4.67% *w*/*v*) (**b**) alginate (% *w*/*v*) and NADES (% *v*/*v*) (CS = 0.2% *w*/*v*).

Based on the RSM plots presented (Figure 7), it can be observed that by maintaining the parameter C (alginate % *w*/*v*) at a constant value of 4.67% *w*/*v*, decreasing the amount of NADES (up to 2% *v*/*v*) while maintaining at the same time higher percentages of CS results in an increased water retention ratio.

#### 2.5.3. Oil Absorption Capacity

The quadratic model, which has an F-value of 13.92, provides the most accurate description of the oil absorption (%) response (R3). Variables with high *p*-values were excluded from the model to enhance its efficacy. The model demonstrates a robust fit, as shown by the R^2^ and adjusted R^2^ values of 0.96 and 0.89, respectively, with a statistically significant model (*p*-value 0.0049). The statistical analysis indicates a signal-to-noise ratio of 12.46, indicating satisfactory signal quality. The statistical significance of each variable is presented in Table 9.

The regression model relating process design parameters A, B, and C to oil absorption (R3) is presented in Equation (3):

R3 = 47.9 − 15.29A − 51.99B − 1.34C + 8.72AB + 0.43AC + 0.2BC + 1.15A^2^ + 20.09B^2^ − 0.22C^2^ (3)

The concentrations of alginate, NADES (ChCl:AA), and chitosan show a statistically significant (*p* < 0.005) effect on the oil absorption capacity of the hydrogels.

**Figure 8:**
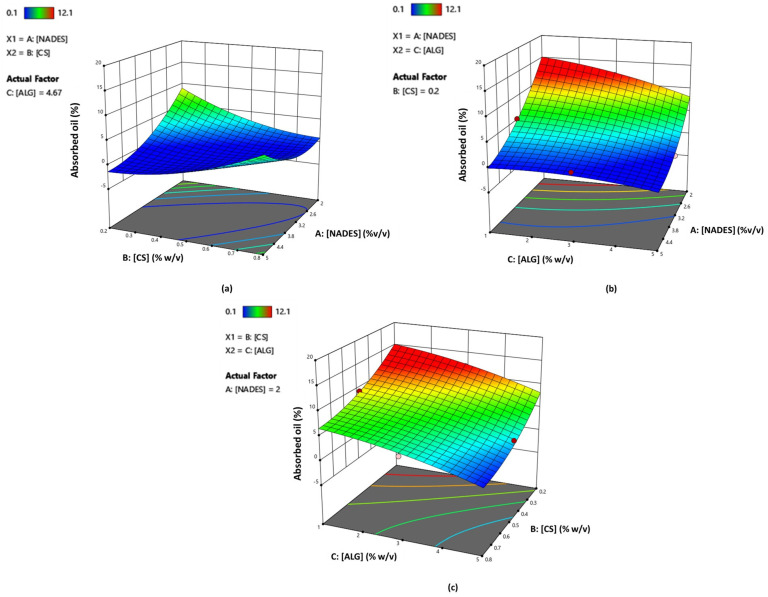
RSM plots for oil absorption (%) response showing the correlation between oil absorption (mg) and the factors: (**a**) CS (% *w*/*v*) and NADES (% *v*/*v*) (alginate = 4.67% *w*/*v*); (**b**) alginate (% *w*/*v*) and NADES (% *v*/*v*) (CS = 0.2% *w*/*v*); (**c**) alginate (% *w*/*v*) and CS (% *w*/*v*) (NADES = 2% *v*/*v*).

Response surface analysis (Figure 8a) shows that in order for a high oil absorption capacity to be achieved (keeping constant the parameter of alginate at 4.67% *w*/*v*), the use of lower concentrations of NADES (2–4% *v*/*v* approximately) in combination with a lower concentration of CS (% *w*/*v*) is needed. Moreover, based on Figure 8b, it is observed that, maintaining a constant value of parameter B (CS% *w*/*v*) at 0.2% *w*/*v*, lower amounts of NADES (2–4% *v*/*v* approximately) in combination with concentrations of alginate in the range of 1–3.5% *w*/*v* result in an elevated oil absorption. Finally, regarding the relationship between parameters B and C (for NADES = 2% *v*/*v*) with respect to the oil absorption capacity, lower percentages of alginate (1–3% *w*/*v*) are necessary when the amount of CS lies in the range of 0.2–0.3% *w*/*v* as defined by the experimental boundaries (Figure 8c).

In this study, the optimization criterion was to maximize all the responses, e.g., the swelling ratio, the water retention ratio, and the oil absorption capacity of the synthesized hydrogels. The following conditions were selected as optimal within the experimental boundaries: 2% *v*/*v* NADES, 0.2% *w*/*v* CS, and 4.67% *w*/*v* alginate. To validate the proposed model, three additional experiments were conducted under the selected optimum conditions, and their results are presented in Table 10. The experimental responses fall within the 95% prediction intervals (PI) according to the estimated model, indicating that the experimental data within the design boundaries fit the model well, as shown in Table 10.

### 2.6. X-Ray Diffraction Analysis (XRD) Analysis

**Figure 9:**
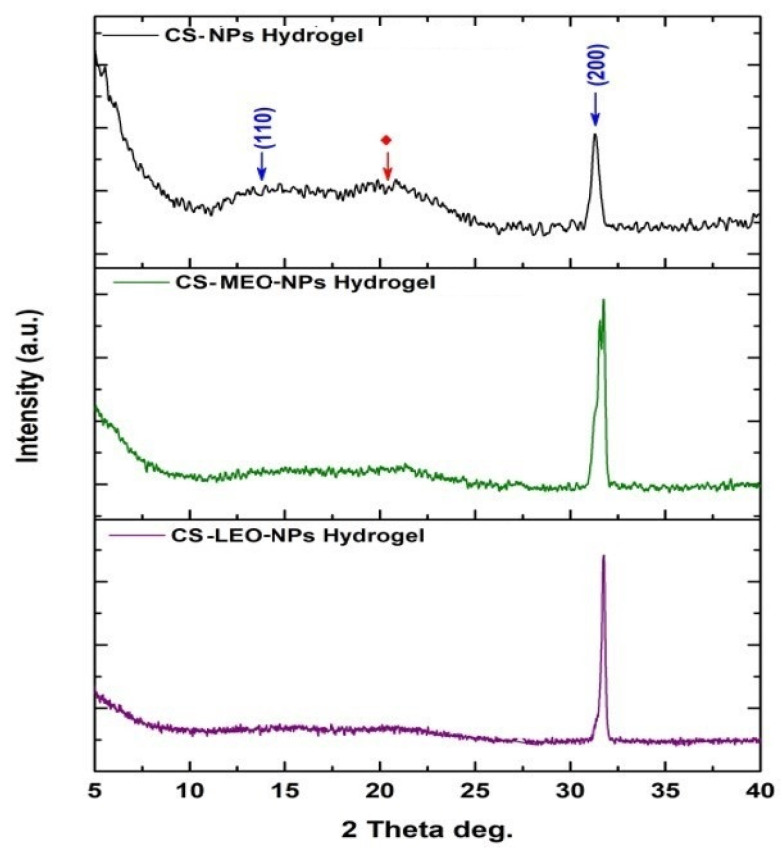
XRD pattern for the prepared hydrogel samples. The crystal planes presented in blue correspond to sodium alginate, while the red symbol is used to indicate the presence of chitosan.

The diffraction diagrams obtained from the X-ray diffraction (XRD) analysis of the hydrogels are illustrated in Figure 9.

According to the obtained data, two diffraction peaks at 2-theta values equal to 13.6° and 30.3° are indexed (marked in blue) for sodium alginate and are attributed to the (110) and (200) planes, originating from the glucuronate and mannuronate units, respectively. Additionally, the broad diffraction peak observed at 2θ value ≈20.5° constitutes a typical fingerprint of semi-crystalline chitosan, corresponding to crystal-II in chitosan structure. The crystallinity index (CI%) of the studied samples was estimated using the following Equation (4):

(4) $$CI\%=\frac{Area\;of\;all\;the\;crystalline\;peaks}{Area\;of\;all\;the\;crystalline\;and\;amorphous\;peaks}$$

Based on the acquired results, the crystallinity indices were calculated as 31.8%, 12.4%, and 11.9% for the CS-NP hydrogel, the CS-MEO-NP hydrogel, and the CS-LEO-NP hydrogel, respectively. It can be concluded that the encapsulation of both essential oils within chitosan NPs leads to the reduction of the hydrogel’s crystallinity, as well as to the enhancement of the intensity of the (200) plane of sodium alginate.

### 2.7. Antimicrobial Studies

The antimicrobial activity of the prepared hydrogels, as well as of pure EOs against foodborne pathogens, are presented in Table 11.

In general, the examined hydrogel systems, as well as the pure EOs, demonstrated moderate antimicrobial activity (diameter 10 < Ø < 14 mm). Notably, in all cases, the antimicrobial activity of hydrogels (except those that incorporated blank CS NPs) was comparable to that of 5% *w*/*v* sodium benzoate. Pure EOs showed higher absolute values of antimicrobial activity when compared to the values of the corresponding hydrogels, while whether these values were accompanied by statistically significant or not differences varied depending on the type of microorganism. More specifically, in cases of E. coli and S. aureus, the alginate hydrogel incorporating CS-LEO-NPs demonstrated (a) statistically significant higher antimicrobial activity when compared to the alginate hydrogel containing CS-MEO-NPs and (b) similar antimicrobial activity with both examined pure EOs. On the other hand, the alginate hydrogel containing CS-MEO-NPs demonstrated higher but not statistically different antimicrobial activity against *L. monocytogenes* when compared to that of the alginate hydrogel incorporating CS-LEO-NPs.

Overall, the findings associated with the antimicrobial potential of the selected systems agree with that of Puvaca et al. [33], who showed that pure LEO produced a 13 mm inhibition zone against S. aureus. Similarly, Ozogul et al. [34] revealed that E. coli, along with *S. paratyphi*, were more vulnerable to French LEO than to eucalyptus and Mentha essential oils. On the other hand, Jovanovic et al. demonstrated that the addition of Mentha essential oil in chitosan films increased the antimicrobial activity against *L. monocytogenes* in cabbage [35].

## 3. Materials and Methods

### 3.1. Materials

Ultra-low molecular weight chitosan (<5 cps, MW: 20,000 avg.) and sodium alginate were supplied from Glentham Life Sciences (Corsham, UK), chlorine chloride from Alfa Aesar (Ward Hill, MA, USA), and L-ascorbic acid from Duchefa Biochemie (Haarlem, The Netherlands). Essential oils were obtained by Pranarom International (Ath, Belgium). Sunflower oil, corn oil, and olive oil were purchased from the local supermarket.

### 3.2. Characterization of NADES

#### Nuclear Magnetic Resonance Spectroscopy (NMR)

Proton (^1^H) NMR spectra are recorded with the 600 MHz Varian instruments (Institute of Chemical Biology, National Hellenic Research Foundation) using deuterated dimethyl sulfoxide (DMSO-d_6_) as the solvent. Chemical shifts are measured in ppm relative to the resonance frequency of tetramethylsilane (TMS). The signal multiplicity is referred to as s (singlet), d (doublet), t (triplet), q (quartet), m (multiplet), br (broad), and coupling constants (*J*) are given in Hz.

### 3.3. NADES Synthesis

The NADES was prepared by mixing choline chloride (ChCl) (5 g, 35.8 mmol) and L-ascorbic acid (AA) (3.15 g, 17.9 mmol) at a molar ratio of 2:1. The ingredients were transferred to a round-bottomed flask equipped with a condenser and heated to 70 °C under an inert atmosphere with continuous magnetic stirring for the time required until a clear liquid formed (about 3 h). The mixture was allowed to cool at room temperature and then stored in a desiccator [36].

^1^H NMR (600 MHz, DMSO-*d_6_*) *δ* 11.002 (s, 1H, H-6), 8.493 (s, 1H, H-7), 5.522 (t, J = 5.2 Hz, 2H, -OH of ChCl), 4.891 (brs, 2H, OH of C-9 and C-10 of ascorbic acid), 4.693 (d, J = 1.6 Hz, 1H, H-8), 3.819 (s, 4H, H-2), 3.706 (brs, 1H, H-9), 3.432–3.409 (m, 2H, H-3), 3.397–3.363 (br, 2H, H-10), 3.378–3.363 (m, 2H, H-10), 3.129 (s, 18H, H-1).

### 3.4. Characterization of Essential Oils

#### Gas Chromatography–Mass Spectrometry Analysis

GC–MS analysis was performed using a Varian (Palo Alto, CA, USA) 220-MS Mass Spectrometer. The column used is Varian VF-5 ms (30 m × 0.25 mm × 0.25 µm). Helium (He) was selected as the carrier gas with a flow rate of 1 mL/min. The inlet temperature was set at 36 °C for 3 min and then increased to 190 °C at 3 °C/min. The method of ionization was Electron Impact and mass analysis was performed using an Ion Trap.

### 3.5. Synthesis of Chitosan Nanoparticles (CS NPs)

An aqueous solution of 2% *v*/*v* ChCl-AA (2:1) was prepared in a round-bottomed flask. Then, 200 mg of CS was added to the NADES solution, and the mixture was left with magnetic stirring for 15 min until CS dissolved completely (final concentration of CS: 0.2% *w*/*v*). Then, the mixture was centrifuged for 15 min at 15,000 rpm to remove impurities. A total of 25 mL of the supernatant was transferred to a round-bottomed flask, and 50 mg of LEO or MEO were added. The mixture was magnetically stirred for 1 h to form the nanoparticles. The nanoparticles’ dispersion was then sonicated at 72 Watts (using Vibra Cell probe sonicator 400 W), was sourced from Sonics & Materials Inc. (Newtown, CT, USA), for 2 min in order to avoid agglomeration.

### 3.6. CS NPs Characterization

#### 3.6.1. Dynamic Light Scattering (DLS)

The measurement of the size, polydispersity index (PdI), and *ζ*-potential of the synthesized nanoparticles was achieved using the Zetasizer Nano ZS instrument was sourced from Malvern Panalytical (Malvern, UK). The NPs (1 mL) were dissolved in ultrapure water and vortexed for 2 min. A type U cell (DTS, Malvern, UK) was used and all assays were performed at room temperature. Each parameter was measured three times, and the average was calculated.

#### 3.6.2. Nanoparticle Tracking Analysis (NTA)

All experiments were conducted with a NanoSight NS300 instrument (Malvern Instruments Ltd., Worcestershire, UK), which was equipped with a 532 nm green laser and a highly sensitive scientific complementary metal–oxide–semiconductor (sCMOS) camera, also from Malvern Instruments Ltd., Worcestershire, UK. For each sample (DS and DP), three independent replicates were analyzed, with three 60-s videos recorded for each replicate. To ensure the validity of the experimental test sample, a minimum of 1000 valid tracks were required. The NTA acquisition was continuously monitored using a syringe loading system. The detection threshold was maintained at four for both labeled and unlabeled particles, and the limiting background noise and camera level settings were kept unchanged between sample acquisitions. The data were processed using NanoSight NTA 3.1 software (Malvern Instruments Ltd., Worcestershire, UK).

#### 3.6.3. Transmission Electron Microscopy Study (TEM)

Transmission Electron Microscopy (TEM) was used to determine the morphological characteristics of CS NPs. TEM is a high-resolution imaging technique utilized for visualizing nanoparticles. The nanoscale investigation was performed with a high-resolution JEOL (Peabody, MA, USA) JEM-2100 LaB6 transmission electron microscope (HRTEM), operating at 200 kV. The samples under investigation were suspended in deionized water and treated with ultrasound to disaggregate the agglomerated particles. A drop from the suspension was then placed on a 300-mesh carbon-coated copper grid and air-dried overnight.

#### 3.6.4. In Vitro Release Study

The dialysis bag method was applied to evaluate the in vitro release of LEO and MEO from the CS NPs. For this method, 5 mL of the aqueous dispersion of NPs (10 mg EO encapsulated in the NPs) was placed in a dialysis bag, which was then added to a beaker that contained 100 mL of phosphate buffer solution with pH = 7.4. The buffer solution was maintained at 37 °C with magnetic stirring at 100 rpm. At predetermined intervals of 20 min, 60 min, 120 min, 180 min, and 240 min, 1 mL aliquots were collected and replaced with fresh media. The concentrations of the main components of essential oil were determined using GC–MS.

### 3.7. Synthesis of Alginate Hydrogel Incorporating the CS-NPs

**Figure 10:**
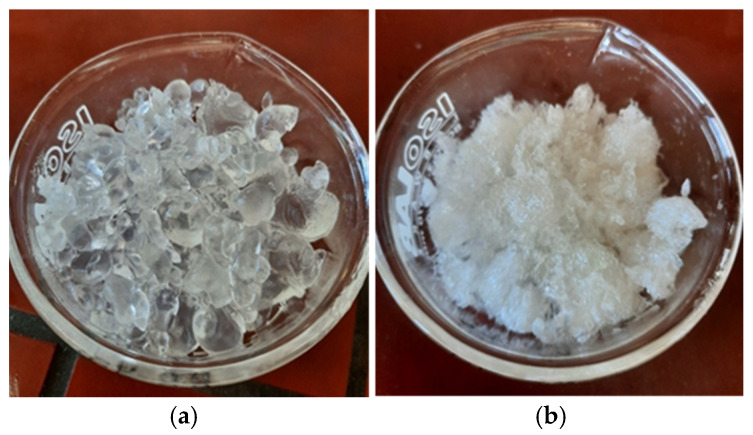
Synthesized hydrogel (**a**) before and (**b**) after freeze-drying.

A solution of sodium alginate 4.67% *w*/*v* in distilled water was prepared. A total of 25 mL of the prepared aqueous sodium alginate solution and 25 mL of CS NPs were added to a crystallizing dish. Ionic crosslinking was achieved with the addition of 10 mL of calcium chloride (CaCl_2_) solution (5% *w*/*v*) dropwise until a homogeneous hydrogel was formed. The synthesized hydrogels were freeze-dried and then stored in a desiccator (Figure 10).

### 3.8. Hydrogel Characterization

#### 3.8.1. Swelling Ratio (SR), Water Retention Ratio (WRR)

In order to calculate the swelling capacity of the synthesized hydrogels, 6 mg from each lyophilized sample was added in 20 mL of 0.1 M hydrochloric acid (HCl) solution (pH 1.2) at 37 °C. The determination of the swelling rate was carried out by weighing the mass of the swollen hydrogels at the time intervals of 5 min, 10 min, 20 min, 40 min, 60 min, 120 min, and 180 min. Mass measurement of the swollen hydrogels was performed after removing the excess aqueous solution from the surface using filter paper.

SR can be calculated by employing the following Equation (5):

(5) $$SR\;(\%)=\frac{Ws-Wd}{Wd}\times 100$$

where *W_s_* and *W_d_* represent the weight of the swollen sample measured each time and the initial weight of the dry sample, respectively.

The water retention ratio is determined by the time point where the hydrogel has reached the equilibrium point. At the equilibrium point, the mass of the hydrogel is kept practically constant. The percentage of water retention is expressed as the percentage of the amount of water retained relative to the equilibrium point and can be calculated by the following Equation (6):

(6) $$WRR\;(\%)=\frac{Ws-Wd}{We-Wd}\times 100$$

where *W_s_*, *W_d_*, and *W_e_* represent the weight of a swollen sample at t time, the initial weight of the dry sample, and the weight of the hydrogel at the equilibrium point, respectively.

#### 3.8.2. Dietary Lipids Absorption Capacity

The capacity of the hydrogel to absorb dietary lipids has been evaluated using an experimental model simulating the pH of the stomach and duodenum tract [25]. In the present study, 200 mg of the lyophilized hydrogel was added to 100 mL of 0.1 N HCl (pH = 1.2) and stirred at 37 °C and 30 rpm, simulating the gastric environment. After 1 h, 3.2 g of oil was added to examine the greatest possible lipid uptake. Three different kinds of oil were used for the experimental study of oil absorption by the hydrogel: Sunflower oil, corn oil, and olive oil. After 30 min, the pH was adjusted to 6.8 by adding dropwise an aqueous NaOH solution (20% *w*/*v*), and the mixture was vigorously stirred for 30 min, mimicking the environment of the duodenum. After 30 min, the hydrogel was removed from the solution, and the external amount of oil and aqueous HCl solution was removed using filter paper. Then, the hydrogel was transferred to a round-bottom flask, where it was rinsed with 30 mL of diethyl ether and stirred at room temperature in order to extract the absorbed oil. Then, the diethyl ether solution was decanted in a round bottom flask and evaporated under reduced pressure. Finally, the absorbed oil was measured gravimetrically.

### 3.9. Optimization of the Preparation Process of the Alginate Hydrogel Incorporating CS-LEO-NPs

Based on the preliminary experiment by a single-factor test, a three-factor and three-level Box Behnken Design (3^3^) experimental design was conducted to optimize the hydrogel preparation using the Design-Expert 12.0 software (Stat-Ease Inc., Minneapolis, MN, USA—Trial Version). Hydrogels were evaluated based on their swelling capacity, water retention capacity, and oil absorption capacity. The parameters of the experimental design are given in Table 12. The concentrations of NADES (A), chitosan (B), and alginate (C) in the hydrogels were employed as the independent variables. The selected responses were the swelling ratio (%) (at 10 min) (R1), the water retention ratio (%) (at 2 h) (R2), and the oil absorption capacity (%) (R3), which are considered to be important factors for the quality of the formed hydrogel.

### 3.10. XRD Analysis

The X-ray diffractometer that was utilized to study the crystallinity of the as-prepared essential oil chitosan NPs loaded alginate hydrogels was a Brucker D8 Advance (Brucker, Bremen, Germany) with a Cu K *α*-radiation (*λ* = 1.5406 A˚) at a voltage of 40 kV and a current of 40 mA. The measurement was conducted at a 2 theta (2*θ*) angle ranging between 5 and 40° with a counting diffraction intensity step of 0.01° per 0.5 s.

### 3.11. Antimicrobial Activity of Hydrogels

The antimicrobial activity of the alginate hydrogels containing essential oils was evaluated by using the disk diffusion method (DDM). More specifically, inocula of standard cultures that were incubated for 24 h at 37 °C in Brain Heart Infusion (BHI) broth (*Escherichia coli*-ATCC 25922, *Staphylococcus aureus*-ATCC 6538, *Salmonella Typhimurium*-ATCC 14028, *Listeria monocytogenes*-ATCC 35152) were then aseptically subjected in 0.9% *w*/*v* sodium chloride (NaCl) at a 0.5 McFarland concentration (10^7^ to 10^8^ CFU/mL). Continuing, 100 µL of the solution was spread with a spatula in Petri dishes containing Mueller–Hinton agar. Meanwhile, freeze-dried essential oil-containing hydrogels were pulverized, while pure essential oils (EOs) were diluted in 2% *v*/*v* dimethyl sulfoxide (DMSO) in order to enhance their solubilization. Hydrogel essential oil-containing powder was then diluted with the corresponding amount of water in order to make final solutions with the same concentration as pure essential oils (i.e., 3.6% *v*/*v*). The hydrogel solutions were vigorously stirred for thirty minutes in order to enhance the dispersion of the EO, while 2% *v*/*v* DMSO was also added. The solutions were kept in a dry and cool place overnight. Finally, sterile filter paper disks (6 mm diameter) were immersed in the tested solutions and were placed on the surface of the inoculated Petri dishes. All samples were tested in triplicate (*n* = 3). Moreover, four types of controls were used: two negative controls with 2% *v*/*v* DMSO as well as NADES and two positive controls such as a widely used food industry preservative (5% *w*/*v* Sodium Benzoate) and one antibiotic (20 µg Ampicillin). In an attempt to reduce the contribution of the solvent, the replacement of 2% *v*/*v* DMSO with a water-ethanol mixture (9:1) was tested. More specifically, the introduction of the aqueous solvent did reduce its contribution to microbial activity but significantly increased the standard deviation between the same measurements, probably due to its inability to fully solubilize the EOs. In all cases, the diameter of the inhibition zone was measured (mm) after incubation of the Petri dishes at 37 °C for 24 h. Since that 2% *v*/*v* DMSO was expected to show a small positive contribution to antimicrobial activity, further data curation was deemed necessary. Thus, by subtracting the value of the inhibition zone of 2% *v*/*v* DMSO (8.3 mm) from that of the disk (6.0 mm), the “fake” contribution of the former was calculated (2.3 mm). In order to evaluate the antimicrobial activity of the samples, we assigned five scales of the diameter measured as follows: Ø < 6 mm, no antimicrobial activity of hydrogel; 6 < Ø < 10 mm, the hydrogel is characterized by low antimicrobial activity; 10 < Ø < 14 mm, the hydrogel demonstrates medium antimicrobial activity; 14 < Ø < 19 mm, the hydrogel exerts good antimicrobial activity; and Ø > 20 mm, the hydrogel shows high antimicrobial activity.

## 4. Conclusions

The aim of the present study was to prepare a hybrid system by incorporating chitosan nanoparticles (CS NPs) loaded with essential oils in alginate hydrogels. The new system is composed of 100% naturally occurring compounds and is considered a step towards greener and safer materials for biomedical and food applications. An additional feature that makes the developed process advantageous is that an aqueous solution of choline chloride/ascorbic acid NADES was used to efficiently dissolve chitosan. This NADES also played the role of the gelation agent for the preparation of chitosan nanoparticles; thus, no addition of another gelation compound was required.

The chitosan nanoparticles encapsulating Mentha and lavender essential oils were characterized using DLS and NTA, while the release rate of the EOs was studied using GC–MS. An indication of the stability of the hydrogel network and the ability of the hydrogels to absorb and retain water was determined. The hydrogels showed significant water retention capacity (>80%) even after the swollen sample was kept in the aqueous HCl solution for 4 h, indicating good stability of the network. The hydrogel incorporating CS-LEO NPs exhibited a high water uptake capacity, surpassing 995 mg H_2_O/mg dry hydrogel after 10 min. Moreover, the hydrogel incorporating the CS-LEO NPs absorbed lipids from sunflower oil in an acidic environment (pH = 1.2) (5.6%). Finally, the EOs and the obtained hybrid hydrogels were evaluated for their antimicrobial activity against foodborne pathogens. Overall, the studied hydrogels exhibited moderate antimicrobial activity, comparable to sodium benzoate 5% *w*/*v*, the widely used food preservative in foods. The promising results obtained show that these biocompatible, green hybrid systems merit further investigation for potential biomedical and food applications.

## Figures and Tables

**Table 1:** Retention times and compound areas.

Peak Number		Retention Time (Min)	Compound	Area (%)
Lavender essential oil (LEO)				
1	20.63	Linalool		26.24
2	24.48	Terpinenol-4		6.00
3	25.23	alpha-Terpineol		1.76
4	27.79	linalyl acetate		40.14
5	29.34	trans-b-ocimene		5.70
6	32.07	Fenchyl acetate		2.41
7	32.65	Geranyl acetate		1.13
8	33.52	Geraniol		1.17
9	35.11	beta-Caryophyllene		2.02
10	41.73	Farnesene		3.98
Total				90.55
Mentha essential oil (MEO)				
1	16.85	limonene		8.00
2	24.36	menthol		83.51
3	29.57	menthyl acetate		8.49
Total				100

**Table 2:** DLS characterization of CS-NPs.

	Hydrodynamic Diameter (nm)	Polydispersity Index (PdI)	ζ-Potential (mV)
CS-NPs	378.0 ± 7.8	0.416 ± 0.043	+19.4 ± 0.3
CS-MEO-NPs	303.7 ± 2.3	0.410 ± 0.063	+20.8 ± 0.4
CS-LEO-NPs	313.3 ± 5.4	0.381 ± 0.098	+23.5 ± 0.2

**Table 3:** NTA results of synthesized CS-NPs.

	Hydrodynamic Diameter (nm)	Concentration (Particles/mL)
CS—NPs (blank)	158.8 ± 2.0	3.89 × 10^7^ ± 3.69 × 10^6^
CS—MEO NPs	130.7 ± 1.7	2.54 × 10^7^ ± 1.68 × 10^6^
CS—LEO NPs	143.4 ± 1.5	3.15 × 10^7^ ± 4.39 × 10^6^

**Table 4:** R^2^ coefficients and equations of each of the release models.

	Zero Order *R*^2^	First Order *R*^2^	Higuchi *R*^2^	Korsmeyer–Peppas *R*^2^	Higuchi Equation	Korsmeyer–Peppas Equation
CS-MEO-NPs	0.9071	0.7279	0.9274	0.9524	*y* = 51.998*x* − 8.229	*y* = 0.8954*x* − 0.0876
CS-LEO-NPs	0.8178	0.4741	0.967	0.9367	y = 40.795*x* + 5.34	*y* = 0.835*x* + 0.14

**Table 5:** Swelling ratio (SR), water retention ratio (WRR), and oil absorption capacity of the prepared hydrogels.

Sample	SR (%) (10 min)	WRR (%) (2 h)	Sunflower Oil Absorption (%)	Corn Oil Absorption (%)	Olive Oil Absorption (%)
Alginate hydrogel incorporating CS-NPs (blank)	212.7 ± 1.7	98.6 ± 0.7	1.5 ± 0.1	1.5 ± 0.4	0.95 ± 0.7
Alginate hydrogel incorporating CS-LEO-NPs	406.7 ± 2.0	99.5 ± 0.1	5.6 ± 0.2	7.0 ± 0.1	3.7 ± 0.3
Alginate hydrogel incorporating CS-MEO-NPs	496.2 ± 2.5	98.6 ± 0.5	2.7 ± 0.3	7.1 ± 0.2	4.8 ± 0.5

**Table 6:** Values of independent (A, B, C) and dependent (R1, R2, R3) variables of CS-LEO hydrogels.

No	Factor A (% *v*/*v*)	Factor B (% *w*/*v*)	Factor C (% *w*/*v*)	R1 (%)	R2 (%)	R3 (%)
1	3.5	0.2	1	365.6	46.6	5.3
2	3.5	0.8	1	607.1	69.7	4.8
3	3.5	0.5	3	600.0	73.2	0.2
4	3.5	0.5	3	450.7	75.7	2.7
5	5	0.5	5	406.1	94.5	0.1
6	2	0.5	5	462.9	97.9	3.4
7	2	0.8	3	170.8	97.6	3.6
8	3.5	0.2	5	509.1	67.1	0.1
9	3.5	0.5	3	552.7	56.3	2.0
10	5	0.5	1	305.5	55.4	0.7
11	2	0.5	1	337.0	80.2	9.2
12	5	0.8	3	200.0	69.0	7.8
13	5	0.2	3	590.7	79.0	0.6
14	3.5	0.8	5	541.9	87.8	0.1
15	2	0.2	3	353.8	84.9	12.1

**Table 7:** Significance of each factor equation model term for the swelling ratio.

Model	Lack-of-Fit	A	B	C	AB	BC	A^2^	B^2^	A^2^B	AB^2^
*p*-value = 0.0110	0.896	0.455	0.053	0.106	0.116	0.115	0.002	0.222	0.003	0.070
F-value = 9.72	0.19	0.65	6.30	3.88	3.61	3.64	34.93	1.95	30.09	5.26

**Table 8:** Significance of each factor equation model terms for the water retention ratio.

Model	Lack-of-Fit	A	B	C	AB	AC	A*^2^*	A^2^B	A^2^C
*p*-value = 0.0054	0.999	0.012	0.012	0.021	0.116	0.134	0.004	0.058	0.339
F-value = 10.25	0.01	12.77	12.43	9.69	3.36	2.99	19.69	5.46	1.07

**Table 9:** ANOVA Table.

Model	Lack-of-Fit	A	B	C	AB	AC	BC	A^2^	B^2^	C^2^
*p*-value = 0.0049	0.587	0.0027	0.6248	0.0053	0.0014	0.0867	0.8460	0.0097	0.0361	0.2312
F-value = 13.92	0.8302	30.53	0.2711	22.23	41.25	4.53	0.0418	16.50	8.08	1.86

**Table 10:** Confirmation experiments (three runs) for the optimization process and predicted values of the selected responses.

Runs	Experimental Swelling Ratio (SR) (%)	Experimental Water Retention Ratio (WRR) (%)	Experimental Oil Absorption (%)
1	405	99	5.7
2	400	98	5.3
3	415	96	5.6
Predicted (%)	451.05	92.07	8.9
95% PI low	254.9	70.4	5.1
95% PI high	647.2	113.7	12.9

**Table 11:** Inhibition Zones (mm).

Hydrogel Type	Inhibition Zone Against * Gram-Negative Bacteria		Inhibition Zone Against * Gram-Positive Bacteria	
	*E. coli* ATC 25922	*S. Typhimurium* ATC 14028	*L. monocytogenes* ATC 35152	*S. aureus* ATC 6538
Alginate hydrogel incorporating CS-LEO-NPs (1 mg/mL)	12.8 ± 1.2 a	12.2 ± 0.8 a	9.6 ± 0.8 a	12.9 ± 0.9 a
Alginate hydrogel incorporating CS-MEO-NPs (1 mg/mL)	11.1 ± 0.4 b	12.2 ± 1.1 a	11.8 ± 1.6 b	11.1 ± 0.4 b
Alginate hydrogel incorporating CS-NPs (blank) (1 mg/mL)	7.5 ± 0.8 c	8.3 ± 0.4 b	8.4 ± 1.4 a	8.6 ± 0.2 c
LEO (3.6% *w*/*v*)	14.7 ± 1.4 a	12.4 ± 0.4 a	12.5 ± 0.5 b	13.5 ± 0.3 a
MEO (3.6% *w*/*v*)	13.2 ± 1.2 a	12.6 ± 0.8 a	12.1 ± 0.8 b	13.7 ± 0.6 a
Sodium Benzoate (5% *w*/*v*)	11.3 ± 0.2 b	11.1 ± 0.4 c	10.2 ± 0.6 a	10.8 ± 0.3 b
Ampicillin (20 μg)	20.0 ± 0.4 d	20.5 ± 0.6 d	19.9 ± 0.3 c	20.1 ± 0.3 d

* Each value is expressed as mean ± standard error (*n* = 3), a–d. Different letters within the same column indicate statistically significant differences at *p* < 0.05.

**Table 12:** Design parameters for Box–Behnken design.

Independent Variables		Level		
Factors		Low (−1)	Medium (0)	High (1)
A	NADES (% *v*/*v*)	2	3.5	5
B	Chitosan (% *w*/*v*)	0.2	0.5	0.8
C	Alginate (% *w*/*v*)	1	3	5
R1		Swelling Ratio (%)		
R2		Water Retention Ratio (%)		
R3		Oil Absorption Capacity (%)		

## Data Availability

Data are contained within the article.
